# Physical education, muscle strengthening exercise, sport participation and their associations with screen time in adolescents

**DOI:** 10.3389/fpubh.2023.1100958

**Published:** 2023-02-20

**Authors:** Xiaoqing Hu, Clemens Drenowatz, Michael Duncan, Ran Bao, Sitong Chen, Jinsheng He, Yan Tang

**Affiliations:** ^1^Center for Post-doctoral Studies of Sport Science, School of Physical Education and Sport Science, Fujian Normal University, Fuzhou, China; ^2^Division of Sport, Physical Activity and Health, University of Education Upper Austria, Linz, Austria; ^3^Centre for Sport, Exercise and Life Sciences, Coventry University, Coventry, United Kingdom; ^4^Active Living Research Program, Hunter Medical Research Institute, New Lambton Heights, NSW, Australia; ^5^Centre for Active Living and Learning, College of Human and Social Futures, University of Newcastle, Callaghan, NSW, Australia; ^6^Institute for Health and Sport, Victoria University, Melbourne, VIC, Australia; ^7^School of Physical Education, Shanghai Normal University, Shanghai, China; ^8^School of Physical Education, Shanghai University of Sport, Shanghai, China

**Keywords:** physical education, muscle activity, sport participation, television viewing time, video time, computer time

## Abstract

**Background/Objective:**

Physical activity (PA) has been suggested to reduce screen time. This study aimed to explore the associations of physical education (PE), muscle-strengthening exercise (MSE), and sport participation with screen time.

**Methods:**

A multi-cluster sampling design was used to select 13,677 school-attending adolescents that participated in the Youth Risk Behavior Surveillance 2019 survey. Adolescents self-reported their frequency of PE attendance, participations in MSE, sport participation and hours for screen time. Additionally, participants provided demographic information including sex, age, race, grade, and weight status.

**Results:**

Collectively, there were beneficial associations between participating in MSE for 4 (OR = 1.31, CI: 1.02–1.68), 5 (OR = 1.65, CI: 1.31–2.08), 6 (OR = 2.23, CI: 1.47–3.36), 7 (OR = 1.62, CI: 1.30–2.01) days and video or computer game hours. Similarly, beneficial associations between participating in 1 team sport (OR = 1.23, CI: 1.06–1.42), 2 team sports (OR = 1.61, CI: 1.33–1.95), 3 or more team sports (OR = 1.45, CI: 1.16–1.83) and video or computer game hours were observed. Participating in 1 team sport (OR = 1.27, CI: 1.08–1.48), 2 teams sport (OR = 1.41, CI: 1.09–1.82), 3 or more team sport (OR = 1.40, CI: 1.03–1.90) also increased the odds for meeting guidelines for television viewing hours. Only 2 days of PE attendance (OR = 1.44, CI: 1.14–1.81) was significantly associated with video or computer game hours.

**Conclusion:**

The promotion of sports participation appears to be an important component for reducing excess screen time in adolescents. Further, MSE may have beneficial effects on reducing time spent on the computer and playing video games.

## Introduction

Detrimental effects of excessive screen time on health outcomes and comprehensive development in adolescents have been well documented (1, 2). Among other key health related outcomes excessive screen time is associated with increased odds of obesity (1, 3), higher risks of depression (4) and anxiety (4), poorer sleep health (5) and lower levels of physical fitness (6). Based on the available evidence, various national public health guidelines recommend that adolescents should not have more than 2 h of screen time per day (7–9). Unfortunately, despite the documented adverse health effects of prolonged screen time of adolescents, the prevalence of adherence to recommended daily screen time is disappointing. Over the past decade, screen time increased in adolescents across the world, particularly due to an increase in computer time (10). Accordingly, an international study including more than 11,000 adolescents indicated that two thirds of adolescents exceeded 2 h of screen time per day (11). The current COVID-19 pandemic along with the implementation of lockdown policies further contributed to an increase in screen time in youth (12–15). In light of this situation, it is necessary to implement actions to limit screen time in adolescents.

A key first step to address excessive screen time is to understand the factors influencing screen time. Various studies have identified correlates of screen time use in adolescents (16–19) based on the Social Ecological Model. According to the Time-Use Epidemiology Framework that has been widely used in physical activity research (20), physical activity and sedentary time are co-dependent behaviors, which suggests that increases in physical activity might reduce sedentary time. Nevertheless, a meta-analysis indicated only a small negative association between physical activity and sedentary time in adolescents (21), which implies that physical activity is probably associated with screen time, as it is a main source of sedentary time (22, 23). It may, however, be necessary to further explore the specific associations between different forms of physical activity and screen time in adolescents. For example, Vella et al. reported that sport participation was associated with adherence to screen time recommendations (no more than 2 h per day) in a nationally representative Australian sample (24). Further, greater engagement in physical education (PE) was associated with less time in front of screen-based devices (25, 26).

Muscle strengthening exercise, a neglected form of physical activity (27), has also received more attention in recent physical activity research. The health benefits of muscle strengthening exercise have been well documented, including a reduction in the odds for depression (28–30), preventing obesity (31), and improving sleep quality (32, 33). Its association with screen time, however, remains unclear. Understanding if and how different types of PA, such as muscle strengthening exercise, might be associated with screen time is a needed step to providing greater nuance and precision in guidance relating to physical activity prescription for health. As yet, this is an underexplored issue. There also remain some gaps regarding the associations of PE or sport participation with screen time. Only a few studies have used nationally representative samples (34) and a majority of previous studies focused on overall screen time rather than differentiating between different kinds of screen time (35–37). To address these research gaps, this study aimed to explore the associations of PE, muscle strengthening exercise, and sport participation with screen time, using the data from 2019 Youth Risk Behavior Surveillance (YRBS), as the YRBS survey provides national data with two kinds of screen time.

## Methods

### Study design and participants

Data from the YRBS 2019, which uses a multi-cluster sampling design to generate a nationally representative sample of youth from 9 to 12 grades, was used for the present analyses. A weight based on student sex, race/ethnicity, and grade was applied to each respondent to adjust for non-response and oversampling of minority students. The overall weights were scaled so that the weighted count of students equals the total sample size and that the weighted proportions of students in each grade match the national population proportions. The YRBS survey has been approved by the Institutional Review Board at the Center for Disease Control of the US. The overall response rate of the 2019 YRBS survey was 60.3%, calculated by the school response rate (75.1%) × the student response rate (80.3%). A total of 13,872 study participants completed the questionnaire in the 2019 YRBS survey, of which 13,677 (50.3% girls) provided valid and complete data.

### Measures

#### Independent variables

In our study, PE attendance, muscle strengthening exercise and sport participation were included as independent variables (1) PE attendance was assessed by the question “In an average week when you are in school, on how many days do you go to PE classes?” with the responses of 0–5; (2) muscle strengthening exercise was assessed with the question “During the past 7 days, on how many days did you do exercises to strengthen or tone your muscles, such as push-ups, sit-ups, or weightlifting?” with the responses of 0–7 days; (3) sport participation was assessed with the question “During the past 12 months, on how many sports teams did you play? (Count any teams run by your school or community groups)” with the responses of 0–3 or more.

#### Outcomes

In the YRBS survey, only television watching hours and video/computer games hours were assessed. Television viewing time was assessed with the question “On an average school day, how many hours do you watch TV?” with the responses of 0-5 or more hours per day and reporting < 2 h was treated as limited television viewing; (3) video or computer games was assessed with the question “On an average school day, how many hours do you play video or computer games or use a computer for something that is not schoolwork? (Count time spent playing games, watching videos, texting, or using social media on your smartphone, computer, Xbox, PlayStation, iPad, or other tablet)” with the same responses and associated categorization of television viewing time measurement. For these two kinds of screen time, no more than 2 h per day of each kind of screen time was viewed as adherence to the screen time recommendation (7).

#### Control variables

Control variables included sex (female or male), age (12 years or younger…18 years or older), grade (9–12), race (white, black or African American, Hispanic/Latino or all other races) and weight status. Overweight or obesity status of participants was determined by body mass index, estimated based on self-reported height and weight, which was subsequently converted to z-scores. In addition, physical activity was included, as assessed by the question “During the past 7 days, on how many days were you physically active for a total of at least 60 min per day? (Add up all the time you spent in any kind of physical activity that increased your heart rate and made you breathe hard some of the time)” with the responses of 0–7 days and reporting 7 days was treated as meeting the well-accepted physical activity guidelines.

### Statistical analysis

All statistical analyses were performed using SPSS 26.0. Missing data were not imputed. The statistical analyses also considered the complex sampling design for nationally representative estimates according to the YRBS. Weighted percentage of each variable was reported with 95% confidence intervals (CI). Binary logistic regression was utilized to assess the associations of independent variables with outcome variables while controlling for sex, age, grade, race, overweight, and obesity as well as physical activity. Adjusted odds ratio with 95% CI was reported to assess the associations of the three independent variables (PE, muscle strengthening exercise and sport participation%) with the respective outcomes. Two separate models were established, of which one was for television watching time and the other one was for video or computer games time. The above statistical analyses were run through the Complex Sample Module in SPSS. Statistical significance as set as *p* < 0.05.

## Results

In total, 13,677 children and adolescents were included in the present study, and the weighted percentage of female participants was 49.4%. Table 1 presents characteristics of the sample. Almost ¾ of the participants (74.1%) were between the ages 15 and 17 years. Besides, there was a relatively even overall distribution of sample size across grades (26.6% in ninth grade, 25.5% in tenth grade, 24.3% in eleventh grade, and 20.8% in twelfth grade). In terms of race, 51.2% of the participants were White. The weighted percentage of overweight and obese were 16.1 and 15.5%, respectively.

**Table 1:** Sample characteristics.

		**n**	**%**	**Weighted %**	**95%CI**	
Total		13,677	100	/	/	/
**Sex**						
	Female	6,885	50.3	49.4	47.9	50.9
	Male	6,641	48.6	50.6	49.1	52.1
	Missing	151	1.1			
**Age group**						
	12 years old or younger	60	0.4	0.3	0.2	0.5
	13 years old	27	0.2	0.1	0.0	0.2
	14 years old	1,699	12.4	11.9	10.9	13.0
	15 years old	3,473	25.4	24.8	23.5	26.0
	16 years old	3,628	26.5	25.6	24.5	26.7
	17 years old	3,102	22.7	23.7	22.5	24.8
	18 years old or older	1,616	11.8	13.7	12.6	14.9
	Missing	72	0.5			
**Grade**						
	9th	3,637	26.6	26.6	25.4	28.0
	10th	3,717	27.2	25.5	24.7	26.3
	11th	3,322	24.3	24.3	23.2	25.4
	12th	2,850	20.8	23.6	22.4	24.8
	Missing	151.0	1.1			
**Race**						
	White	6,668	48.8	51.2	46.4	56.0
	Black or African American	2,040	14.9	12.2	10.2	14.6
	Hispanic/Latino	3,038	22.2	26.1	21.8	30.9
	All other races	1,493	10.9	10.5	7.9	13.9
	Missing	438	3.2			
**Overweight**						
	Yes	1,933	14.1	16.10	14.90	17.50
	No	10,207	74.6	83.90	82.50	85.10
	missing	1,537	11.2			
**Obesity**						
	Yes	1,795	13.1	15.50	13.80	17.30
	No	10,345	75.6	84.50	82.70	86.20
	missing	1,537	11.2			

The prevalence of different kinds of movement behaviors are outlined in the Table 2. Notably, only 23.2% of included participants reported sufficient physical activity. The percentage of children and adolescents who spent more than 2 h per day watching television and playing video games or using a computer were 80.2 and 53.9%, respectively. Moreover, 47.8% of children and adolescents did not participate in any PE in the previous week. The prevalence of 1, 2, 3, 4, 5 days with PE were 3.2, 5.2, 13.8, 4.1, and 25.9%, respectively. In addition, the weighted percentage of participants who did not participate in muscle strengthening exercise was 29.7%. The highest prevalence of participating in muscle strengthening exercise was reported for 7 days (12.8%), while the lowest prevalence was reported for 6 days (4.4%). Almost half of the sample (42.6%) did not participate in any team sport and 25.9 and 18.2% participated in one and two teams, respectively. Only 13.3% of the sample participated in 3 or more teams.

**Table 2:** Prevalence of different independent and outcomes included in this study.

		**n**	**%**	**Weighted %**	**95%CI**	
Total		13,677	100.0	/	/	/
**Sufficient physical activity**						
	Yes	2,954	21.6	23.2	21.9	24.6
	No	10,266	75.1	76.8	75.4	78.1
	Missing	457	3.3			
**Television watching hours**						
	Yes	10,200	74.6	80.2	78.7	81.7
	No	2,596	19.0	19.8	18.3	21.3
	Missing	881	6.4			
**Played video or computer games or used a computer**						
	Yes	7,246	53.0	53.9	52.1	55.6
	No	5,931	43.4	46.1	44.4	47.9
	Missing	500	3.7			
**Physical education attendance days**						
	0 days	5,865	42.9	47.8	42.6	53.1
	1 day	346	2.5	3.2	2.2	4.5
	2 days	534	3.9	5.2	3.2	8.3
	3 days	1,344	9.8	13.8	9.3	20.0
	4 days	364	2.7	4.1	2.6	6.4
	5 days	2,835	20.7	25.9	21.5	31.0
	Missing	2,389	17.5			
**Muscle strengthening exercise days**						
	0 days	2,568	18.8	29.7	27.6	32.0
	1 day	812	5.9	9.2	8.4	10.2
	2 days	973	7.1	11.6	10.8	12.5
	3 days	1,067	7.8	12.6	11.3	14.1
	4 days	763	5.6	9.1	8.4	9.9
	5 days	877	6.4	10.5	9.4	11.8
	6 days	361	2.6	4.4	3.8	5.1
	7 days	1,053	7.7	12.8	11.9	13.8
	Missing	5,203	38.0			
**Sport participation**						
	0 teams	4,242	31.0	42.6	39.6	45.7
	1 team	2,584	18.9	25.9	24.7	27.0
	2 teams	1,737	12.7	18.2	16.8	19.7
	3 or more teams	1224	8.9	13.3	11.4	15.4
	Missing	3,890	28.4			

**Figure 1:**
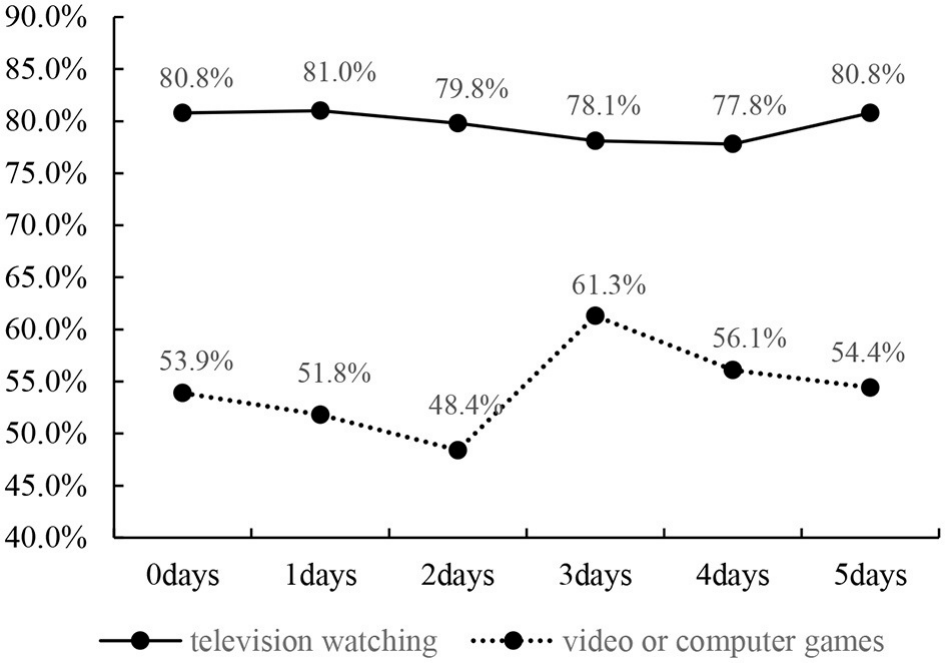
Prevalence of no more than 2 h per day of television watching and video or computer by physical education attendance days.

The prevalence of no more than 2 h per day of television watching and video or computer games by PE attendance days are shown in the Figure 1. The same prevalence (80.8%) of no more than 2 h per day of television watching was reported for 0 day and 5 days. Moreover, the prevalence of no more than 2 h per day of television watching decreased as the number of PE attendance increased (from 1 day to 4 days). No such trend was observed for time spent at the computer or playing video games. The included children and adolescents who participated in PE for 2 days reported the highest prevalence of no more than 2 h per day of video or computer games (61.3%), and the lowest prevalence is 48.4% for participating in PE for 3 days.

**Figure 2:**
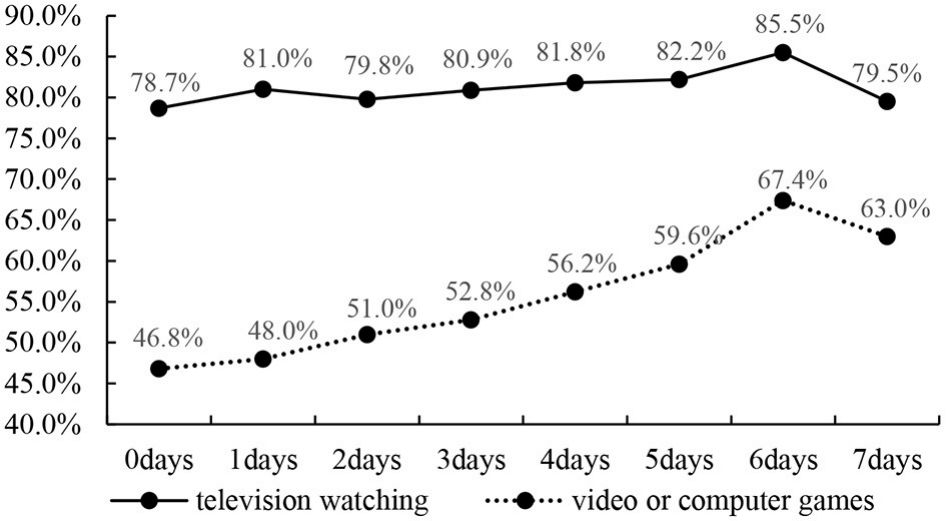
Prevalence of no more than 2 h per day of television watching and video or computer games by muscle strengthening exercise days.

Figure 2 display the prevalence of no more than 2 h per day of television watching and video or computer games by muscle strengthening exercise days. Overall, the prevalence of no more than 2 h per day of video or computer games increased with the number of days participants engage in muscle strengthening exercises, except for 7 days. The highest prevalence of no more than 2 h per day of video or computer (67.4%) therefore, was observed with 6 days of muscle strengthening exercise. Comparable results were observed for the prevalence of no more than 2 h per day of television watching, with an increasing prevalence from 2 days to 6 days. The highest prevalence was 85.5% for 6 days.

**Figure 3:**
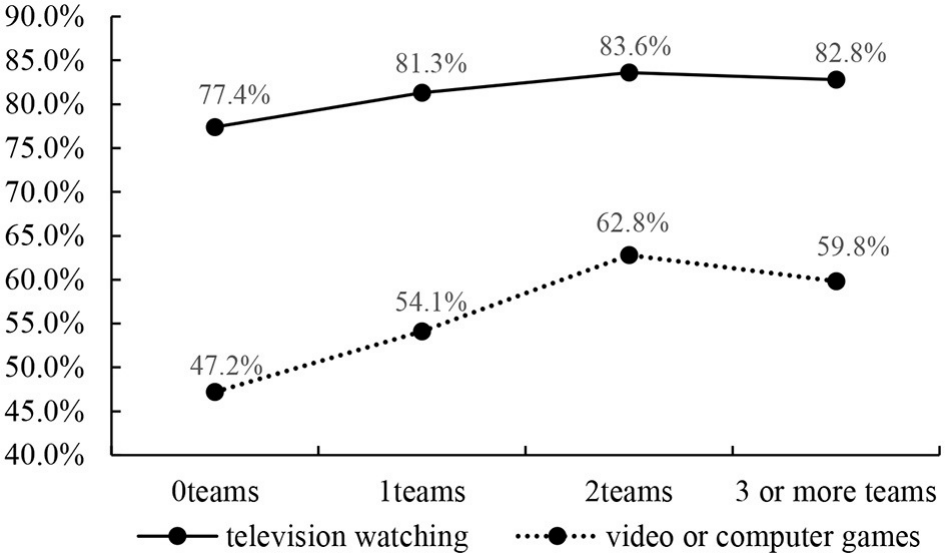
Prevalence of no more than 2 h per day of television watching and video or computer games by sport participation.

Figure 3 show the prevalence of no more than 2 h per day of television watching and video or computer games by sport participation. The included study participants who participated in more team sports had a higher prevalence of no more than 2 h per day of television watching, except for participation in 3 or more team sports. Similar results were found for the prevalence of no more than 2 h per day of video or computer games by sport participation. The highest prevalence of no more than 2 h per day of television watching and video or computer games was 83.6 and 62.8%, respectively for participants reporting being on two teams.

The association between PE, muscle strengthening exercise and sport participation with screen time is presented in Table 3. Only 2 days of PE attendance was significantly associated with video or computer game hours (no more than 2 h per day; OR = 1.44, CI: 1.14–1.81). Further, there were beneficial associations between participating in muscle strengthening exercise for 4 (OR = 1.31, CI: 1.02–1.68), 5 (OR = 1.65, CI: 1.31–2.08), 6 (OR = 2.23, CI: 1.47–3.36), 7 (OR = 1.62, CI: 1.30–2.01) and video or computer game hours (no more than 2 h per day). Additionally, participating in 1 team sport (OR = 1.23, CI: 1.06–1.42), 2 teams sport (OR = 1.61, CI: 1.33–1.95), 3 or more teams sport (OR = 1.45, CI: 1.16–1.83) increased the odds reporting no more than 2 h per day of video or computer games. Team sports participation was also associated with television viewing hours. Specifically, there were beneficial associations between participating in one team sport (OR = 1.27, CI: 1.08-1.48), 2 team sports (OR = 1.41, CI: 1.09-1.82), 3 or more team sports (OR = 1.40, CI: 1.03-1.90) with television viewing hours (no more than 2 h per day).

**Table 3:** Associations of physical education, muscle strengthening exercise days and sport participation with screen time.

	**Television viewing hours (no more than 2 h per day)**			**Video or computer game hours (no more than 2 h per day)**		
	**OR**	**95%CI**		**OR**	**95%CI**	
**Physical education attendance days** **(ref** = **0 days)**						
1 day	1.06	0.71	1.58	0.85	0.66	1.10
2 days	0.83	0.58	1.20	**1.44**	**1.14**	**1.81**
3 days	0.89	0.70	1.13	0.78	0.65	0.93
4 days	0.80	0.49	1.31	1.10	0.65	1.86
5 days	0.92	0.78	1.10	0.89	0.75	1.07
**Muscle strengthening exercise days** **(ref** = **0 days)**						
1 day	1.09	0.76	1.55	1.10	0.88	1.37
2 days	0.84	0.63	1.12	1.14	0.95	1.36
3 days	0.82	0.65	1.03	1.19	1.00	1.42
4 days	0.82	0.59	1.15	**1.31**	**1.02**	**1.68**
5 days	0.90	0.66	1.23	**1.65**	**1.31**	**2.08**
6 days	0.79	0.49	1.28	**2.23**	**1.47**	**3.36**
7 days	0.65	0.45	0.95	**1.62**	**1.30**	**2.01**
**Sports team participation** **(ref** = **0 teams)**						
1 team	**1.27**	**1.08**	**1.48**	**1.23**	**1.06**	**1.42**
2 teams	**1.41**	**1.09**	**1.82**	**1.61**	**1.33**	**1.95**
3 or more	**1.40**	**1.03**	**1.90**	**1.45**	**1.16**	**1.83**

Ref, reference group; OR, odds ratio; CI, confidence interval; Bold font, statistically significant.

## Discussion

This study explored the associations of different forms of physical activity (PE, muscle strengthening exercise, team sports) with two kinds of screen time (television watching hours and video/computer games hours) in adolescents using the 2019 YRBS data. After controlling for physical activity and some selected sociodemographic factors, there was a beneficial association of sports participation with time spent watching television and playing video/computer games. Further, engaging in at least 4 days of muscle strengthening exercise was associated with less time spent playing video/computer games. Associations between PE and screen time in adolescents, however, were not evident based on our results.

Sports participation, as a form of physical activity, was consistently associated with lower risks of excessive screen time (more than 2 h per day), including watching television and video/computer games. These findings are in line with previous studies (24, 36, 38). For example, a study in Spanish adolescents found that sport participation was linked with lower odds for more than 4 hours of total screen time (36). Similarly, Vella et al. (24) found that higher levels of sport participation were associated with a greater likelihood to meet the screen time recommendations (< 2 h per day) in Australian adolescents. Although these studies examined total screen time, the accumulated evidence indicates beneficial associations between sports participation and screen time in adolescents, which provides theoretical base that sports participation could reduce specific kinds of screen time. A possible explanation is that sports participation may take place at times when opportunities for screen time are also high, such as during after-school period (24).

The results of the present study, however, only showed a limited dose-response relationship between sports participation and screen time, which is in contrast to a previous study by Sirard et al. (38). This difference may be attributed to methodological differences in recruiting study participants or measures to assess sport participation and screen time. Due to the differences between our study and Sirard et al. (38), it is currently impossible to establish a causal association between sports participation and screen time in adolescents. Accordingly, further research is needed to address this issue. As this study cannot elucidate the underlying mechanism linking sport participation with specific types of screen time future research should also tackle this interesting question. In addition, associations between sports participation and other types of screen time, such as educational screen time or social-oriented screen-based time need to be explored. Such additional research would be beneficial to comprehensively understand the associations between sports participation and various kinds of screen time in adolescents.

Regarding muscle strengthening exercise, four or more days of engagement in this form of physical activity was associated with a larger probability of engaging in < 2 h per day of video/computer games in adolescents. To our knowledge, very few previous studies examined this association and, as a consequence, the current study presents new insight in this regard. As has been addressed in relation to sport participation, muscle strengthening exercise may be predominantly performed during leisure time, which would minimize the available time to engage in sedentary pursuits such as playing computer or video games. It may, however, be possible to watch television, while engaging in resistance exercise, which could explain the limited association between television time and engagement in muscle strengthening activities. Given the potential benefits of muscle strengthening exercise with screen time additional research in this area is warranted. This may also allow researchers and practitioners to better understand and unpick the limited association between muscle strengthening exercise and television time, which is critical for the development of intervention strategies targeting overall screen time in adolescents.

Interestingly, the results of the present study did not show an association between PE and time spent watching television. The limited benefits of PE on screen time in the present study may be attributed to the quality of PE, which potentially fails to enhance health literacy and a lack of education on the potential health risks of excess screen time in adolescents. Nevertheless, participation in PE has been associated with increased physical activity (39, 40) and there is also research indicating beneficial associations between PE and screen time. Chen et al. (25), for example, showed less sedentary time on days with PE. Similar results were also shown in an international study with 9–11-year-old children from 12 countries (26). Accordingly, it can be argued that a higher amount of PE reduces objectively measured daily sedentary behavior. As screen time is a major source of sedentary behavior, it could be expected that PE can have beneficial effects on screen time in adolescents. These studies, however, focused on physical activity during PE and the association with sedentary time, while the present study considered days for PE attendance. The reliance on self-report rather than objective measures, as in the previously cited studies (25, 26), may also contribute to the differences in the results in the present study, in comparison to prior work. It should also be considered that even though screen time is a main source of sedentary behavior, different measures of independent and outcomes probably result in two discrepant associations in adolescents. The importance of PE, nevertheless, has been emphasized by the Society of Health and Physical Educators America (41), as it fulfills the responsibility to promote an active lifestyle and discourages sedentary behaviors in adolescents. Additional research, that examines associations between specific contents and methodologies used in PE with differential health-related outcomes, including various components of sedentary behavior, is warranted to enhance the understanding of the role of PE in health promotion in youth.

## Study limitations and strengths

There are also some study limitations that must be acknowledged. First, the use of self-report questionnaires has a risk of recall bias and social desirability of study participants. However, it should be acknowledged that collation of data of this nature in large representative samples is difficult unless self-report measures are used. Secondly, the cross-sectional study design does not allow to determine casual associations between physical activity contexts and screen time. Further, only frequency of participation in different forms of physical activity was reported with no information on time spent in the respective activities. Despite these limitations, the study has several strengths that are worthwhile mentioning. The use of a nationally representative sample increases the generalisability of the research findings. Furthermore, the present study is one of very few to explore the association between muscle strengthening exercise and screen time in adolescents despite the acknowledgment that muscle strengthening exercise is important for health in youth (28–30).

## Conclusion

This study examined the associations between different forms of physical activity and screen time in adolescents. The results highlight the potential benefits of sports participation in addressing excess screen time (television watching and video/computer) in adolescents. Additionally, engagement in muscle strengthening exercise appears to have beneficial associations with screen time in adolescents, particularly regarding time spent playing computer/video games, while associations of screen time with PE attendance were limited. Additional research, however, is necessary to enhance our understanding of the complex relationship between various components of physical activity and different components of screen time in adolescents in order to inform the design and implementation of efficient interventions aiming to reduce screen time.

## Data availability statement

Publicly available datasets were analyzed in this study. This data can be found at: Youth Risk Behavior Surveillance.

## Ethics statement

The YRBS survey has been approved by the Institutional Review Board at the Centre for Disease Control of the US.

## Author contributions

XH, JH, and YT contributed to performing data analysis, drafting the manuscript, and the conception and design of the study. CD, MD, RB, and SC reviewed and revised the manuscript. All authors contributed to the manuscript revision, read, and approved the submitted version.
